# Supplementary material for: Integrated Hypoxia Signaling and Oxidative Stress in Developmental Neurotoxicity of Benzo[a]Pyrene in Zebrafish Embryos
Source: Antioxidants (Basel). 2020 Aug 11;9(8):731. doi: 10.3390/antiox9080731 (PMC7464806; doi:10.3390/antiox9080731)
Supplement: Supplementary file 1 [file antioxidants-09-00731-s001.pdf]

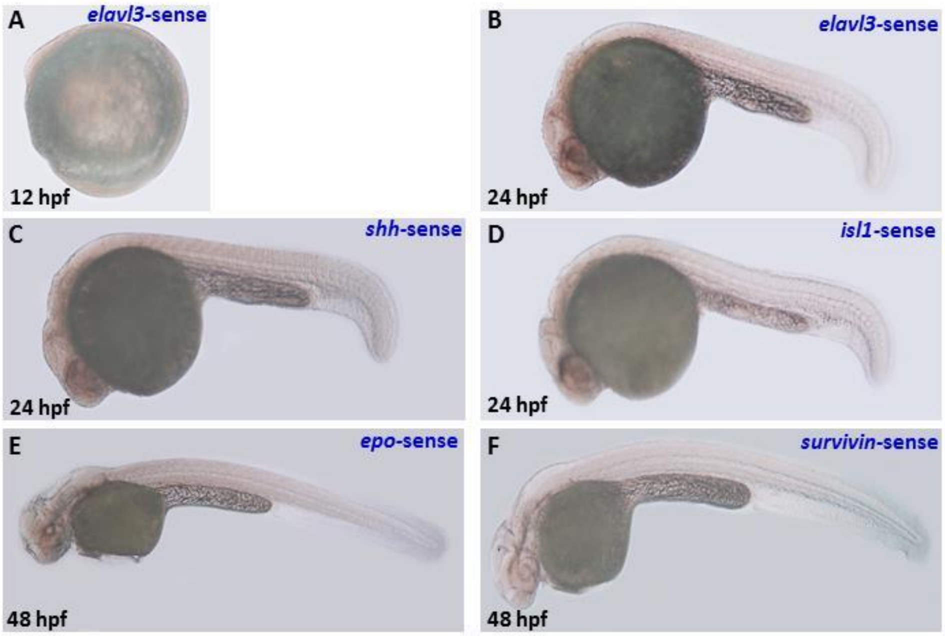

Figure S1. Sense riboprobe serves as negative controls for in situ hybridization staining assy.

In situ hybridization staining with gene-specific sense riboprobe was preformed to show the specificity of (A, B) *elavl3* in 12 and 24 hpf embryos; (C) *shh* and (D) *isl1* in 24 hpf embryos; and (E) *epo* and (F) *survivin* in 48 hpf. No background staining signal was presented in all embryos.
